# 360-degree cervical spinal arthrodesis for treatment of pediatric cervical spinal tuberculosis with kyphosis

**DOI:** 10.1186/s12891-016-1034-7

**Published:** 2016-04-23

**Authors:** Hao Zeng, Xiongjie Shen, Chengke Luo, Zhengquan Xu, Yupeng Zhang, Zheng Liu, Xiyang Wang, Yong Cao

**Affiliations:** Department of Spine Surgery, the Xiangya Hospital of Central South University, 87# Xiangya Road, Changsha, Hunan 410008 People’s Republic of China; Department of Spine Surgery, Hunan Provincial People’s Hospital, Changsha, Hunan 410005 People’s Republic of China

**Keywords:** 360-degree arthrodesis, Cervical spinal tuberculosis, Children, Combined posterior and anterior approaches, Kyphosis

## Abstract

**Background:**

There is limited evidence to guide treatment for pediatric cervical spinal tuberculosis with kyphosis (PCSTK). This study retrospectively evaluates the safety, feasibility and efficacy of 360-degree arthrodesis combined with anterior debridement and decompression for treating PCSTK, while simultaneously emphasizing the role of posterior fixation for the correction and maintenance of the kyphosis angle.

**Methods:**

From May 2006 to December 2012, a total of 12 children with PCSTK underwent 360-degree cervical spinal arthrodesis followed by debridement of focus and decompression of the spinal cord. Data on the angle of kyphosis correction, visual analogue scale scores of pain, the American Spinal Injury Association scoring system of nerve function scores, erythrocyte sedimentation rate (ESR) and body weight were collected at certain periods. Clinical efficacy was evaluated by statistical analysis based on collected data.

**Results:**

Average follow-up period was 34.3 ± 8.6 months. No postoperative complications related to the instrumentation occurred, and neurologic function improved in various degrees. Preoperative kyphosis angle was 41.4 ± 5.2°, and significantly decreased to -4.9 ± 4.9° after surgery. The correction of kyphosis and loss of correction were 47.1 ± 4.9° and 0.6 ± 1.4°, respectively. Average pretreatment ESR was 49.8 ± 13.2 mm/h, which normalized (8.5 ± 0.6 mm/h) within three months in all patients. Average preoperative visual analogue scale was 6.6 ± 1.6, which decreased to 2.3 ± 1.4 postoperatively and 0.3 ± 0.5 during the final follow-up. Mean preoperative body weight was 25.9 ± 5.1 kg, and body weight was 33.5 ± 4.8 kg at the third month of post-operation. Bone healing was achieved in all patients after a mean period of 5.4 months.

**Conclusions:**

360-degree arthrodesis combined with anterior debridement and decompression is a safe and effective method for the treatment of PCSTK. For the correction and maintenance of the kyphosis angle, additional posterior fixation is recommended.

## Background

Cervical spinal tuberculosis (CST) accounts for 3–5 % of spinal tuberculosis. Pediatric CST is less common, but produces more severe morbidities with patient growth. Since few studies focus on pediatric CST, treatment of the disease remains controversial [1–4].

Different from adult cervical spine, pediatric cervical spine has unique anatomical characteristics with growth potential. The growth potential of the anterior vertebral body is most likely interfered by tuberculosis. With normal growth of the posterior elements, cervical spinal deformities will occur. The disease will become worse with presence of focus and nerve compression, which require surgical intervention. Various surgical methods have been reported in the treatment of thoracic and lumbar spinal tuberculosis, but there are few literatures that describe how to surgically address PCSTK. Upadhyay et al. [5] suggested anterior debridement plus autograft/allograft bone reconstruction in a comparative analysis of the short and long-term results of two surgical procedures. They concluded that anterior radical surgery is better than pure debridement surgery for the improvement of the deformity angle. Other authors [2, 6–8] advocated anterior plate combined with debridement, decompression and titanium mesh cage or autograft/allograft bone. Indeed, anterior surgery offers a most direct approach for adequate decompression and effective stabilization and reconstruction of the cervical spine. However, a stand-alone anterior approach is just confined in focusing on immediate interests, which is ignorant of the fact of the disproportionate growth potential between anterior and posterior elements in the long term. Therefore, some authors [9–12] recommend an additional posterior instrumentation to impede posterior growth for balance. Most importantly, apart from this, posterior fixation plays an irreplaceable role in the correction and maintenance of kyphosis for the treatment of CST.

There are limited studies on the treatment of PCSTK, and current surgical techniques remain controversial. This study aims to evaluate the safety, feasibility and efficacy of single-stage 360-degree cervical spinal arthrodesis combined with anterior debridement and decompression for the treatment of pediatric cervical spinal tuberculosis with kyphosis.

## Methods

### Basic information

**Fig. 1:**
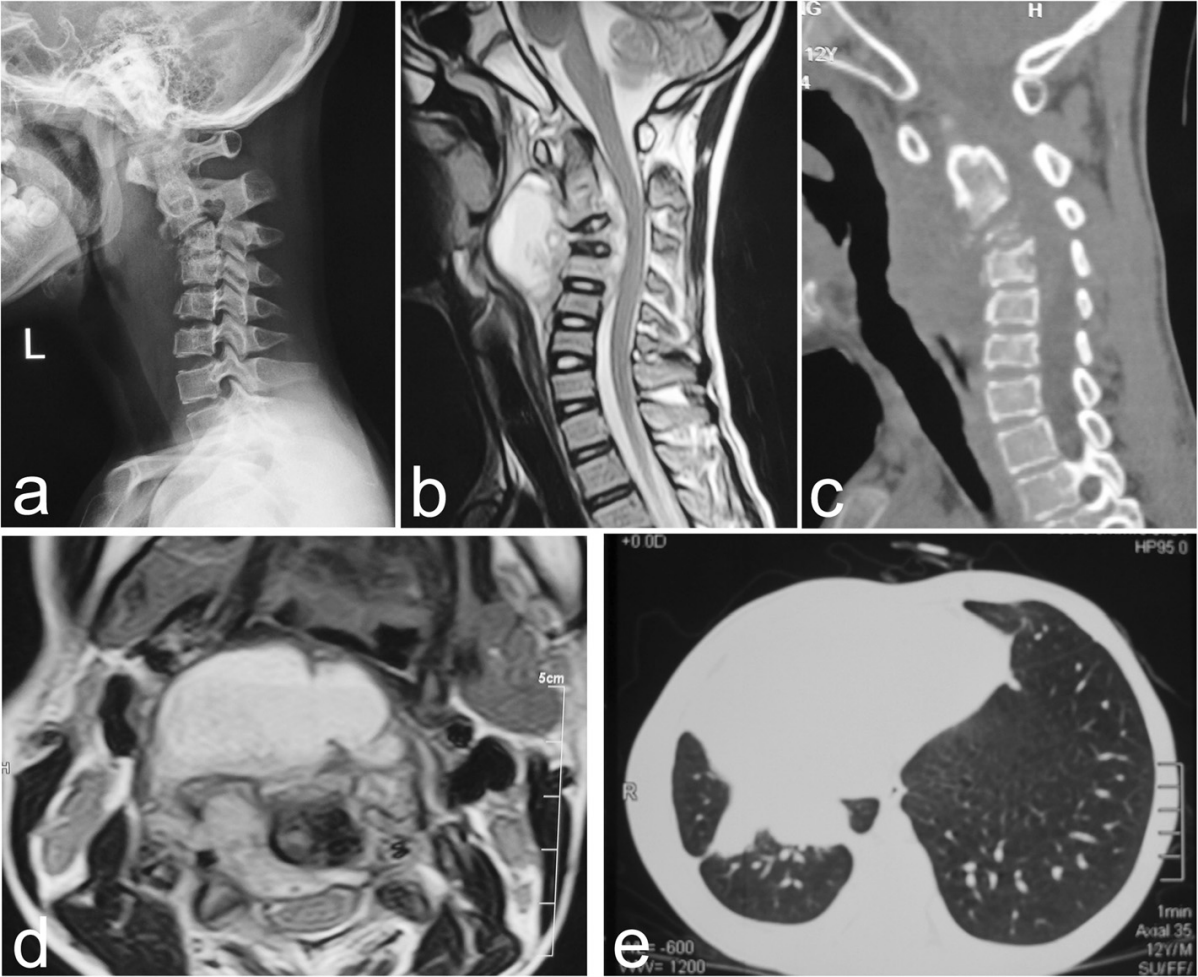
Preoperative images of patient no. 5. (**a**) Lateral X-ray of a 12-year-old male child demonstrated vertebral body damage and sagittal instability and kyphosis. A preoperative sagittal MRI (**b**) and CT (**c**) revealed significant C3-4 vertebral body destruction with kyphosis associated with epidural, paravertebral and retropharyngeal abscess formation; and the cervical spinal cord was severely compressed. Coronary MRI (**d**) revealed a huge abscess that affected the esophagus and invaded into the spinal canal. (**e**) CT scan and enhancements of the lung: the right thorax collapsed and pleural thickening occurred, suggesting tuberculous pleurisy and encapsulated pleural effusion

**Fig. 2:**
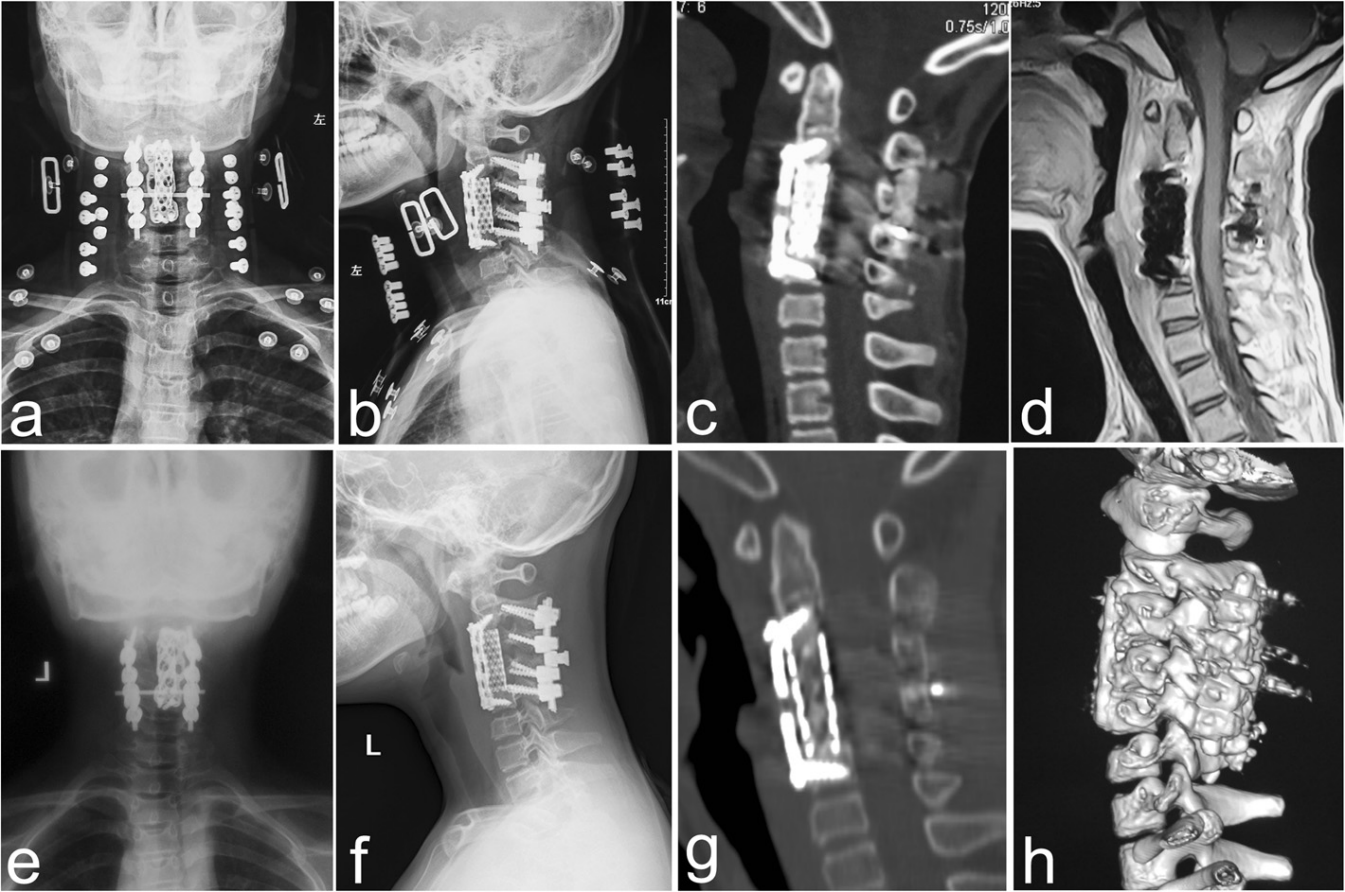
Postoperative and follow-up images of patient no. 5 are shown. (**a** and **b**) Postoperative anterior-posterior and lateral X-ray indicate that the kyphosis significantly improved using one-stage 540-degree fusion combined with anterior debridement and decompression. Sagittal CT (**c**) and MRI (**d**) revealed satisfactory focal clearance and decompression without graft and instrumentation-related complications and relapse of Pott’s disease at three months postoperation. (**e** and **f**) Anterior-posterior and lateral X-ray revealed that the screws, rods, plate and TMC was in good shape without fractures and displacement at 12 months postoperation. Sagittal CT-scan (**g**) and three-dimensional reconstruction (**h**) revealed continuous bridging, and the bone trabeculae at the graft-host vertebral endplate junction had no pseudoarthrosis formation; indicating the superb maintenance of correction of kyphosis at the final follow-up

Written informed consent was obtained from all guardians of patients. This study protocol was approved by the Ethics Committee of Xiangya Hospital. From May 2006 to December 2012, 60 patients diagnosed with CST underwent surgery at our spinal center, including 12 patients who suffered from PCSTK. Among these 12 patients, seven were male and five were female; and patient age ranged from 7–15 years (average age, 10.6 ± 2.6 years). Clinical details of the surgical group are presented in Tables 1 and 2. The diagnosis of tuberculosis for PCSTK was guided by non-specific laboratory findings such as anemia, hypoproteinemia and elevated ESR, as well as by radiological findings including spinal X-ray images, computed tomography (CT) and magnetic resonance imaging (MRI, Figs. 1 and 2). All patients presented with neck pain, nuchal rigidity/restricted neck activity/torticollis, as well as anorexia and weight loss. However, constitutional symptoms including night sweats and mild fever could only be seen in four cases, accounting for 33 % of all patients; while cervical radiculopathy was found in eight cases (67 %), spastic quadriparesis was found in seven cases (58 %), and pulmonary tuberculosis and/or tubercular pleurisy/hydrothorax was found in three cases (25 %, Table 1). All children suffered from severe local or gross kyphosis with a deformity angle of more than 30°. Although retropharyngeal abscess of different sizes were present in all patients, symptomatic dysphagia or dyspnea occurred in only three cases. The American Spinal Injury Association scoring system (Table 2) was used to assess neurological function; in which two cases were classified as grade B, three cases were classified as grade C, two cases were classified as grade D, and five cases had normal neurological function. Body weight and ESR of patients upon admission ranged from 20 to 35 kg and from 23 to 68 mm/h, with an average of 25.9 ± 5.1 kg and 49.8 ± 13.2 mm/h, respectively.

**Table 1:** Clinical presentation on admission

Clinical features	Number of patients (%)
Neck pain	12(100 %)
Nuchal rigidity/restricted neck activity/torticollis	12(100 %)
Anorexia and weight loss	12(100 %)
Cervical radiculopathy	8(67 %)
Spastic quadriparesis	7(58 %)
Mild fever and/or perspiration	4(33 %)
Dysphagia or dyspnea by retropharyneal abscess	3(25 %)
Pulmonary tuberculosis and/or tuberculous pleuritis	3(25 %)

**Table 2:** Clinical details of the surgical group

Patient No	Affected level	Fusion Level	Body weight (kg)		Follow-up(mon)	ASIA			Kyphosis angle (°)			VAS			ESR	
			Pre	Post 3mon*		Pre	Post	FFU^※^	Pre	Post	FFU^#^	Pre	Post	FFU^¥^	Pre	Post 3mon^§^
1	C4-C5	C3-C6	28	36	30	E	E	E	40	-5	-4	7	3	0	66	8
2	C3-C4	C2-C5	24	34	26	E	E	E	44	-4	-4	8	5	0	46	7
3	C4-C6	C3-C7	20	28	30	C	E	E	37	-10	-9	5	1	0	45	9
4	C5-C6	C4-C7	22	30	48	B	D	E	40	-10	-8	4	0	0	68	10
5	C3-C4	C2-C5	28	38	28	D	E	E	35	-9	-9	7	4	0	43	7
6	C4-C6	C3-C7	20	33	30	E	E	E	42	-2	-1	6	2	0	49	9
7	C5-C6	C4-C7	27	32	36	C	E	E	55	1	3	6	1	1	38	4
8	C4-C6	C3-C7	33	40	48	B	D	D	40	0	-2	8	2	1	59	13
9	C4-C6	C3-C7	20	25	37	C	E	E	40	-10	-9	9	3	1	41	8
10	C5-C6	C4-C6	24	29	46	E	E	E	38	-11	-10	4	1	0	60	10
11	C3-C4	C3-C4	35	37	24	D	E	E	46	0	-2	8	3	0	60	8
12	C5-C6	C4-C6	30	40	29	E	E	E	40	1	3	7	2	0	23	9
Mean values			25.9 ± 5.1	33.5 ± 4.8	34.3 ± 8.6				41.4 ± 5.2	-4.9 ± 4.9	-4.3 ± 4.7	6.6 ± 1.6	2.3 ± 1.4	0.3 ± 0.5	49.8 ± 13.2	8.5 ± 0.6

*ASIA* American Spinal Injury Association (ASIA) score system of nerve function, *VAS* Visual Analogue Scale (VAS) scores of pain, *ESR* Erythrocyte Sedimentation Rate, *Pre, Post, FFU* preoperative, postoperative, final follow-up, *Mon* months

^*, §^: Analyzed by paired *t*-test, postoperative at three months compared with preoperative, *P* = 0.001; 0.000

^#, ¥^: Analyzed by paired t test, final follow-up compared with preoperative, *P* = 0.000; 0.000

^※^: Wilcoxon signed-rank test, final follow-up compared with preoperative, *P* = 0.01

### Preoperative procedure

Patients that participated in this study were clinically diagnosed with PCSTK and received HRE [isoniazid (H), rifampicin (R), ethambutol(E)] chemotherapy regimen for 2–4 weeks prior to surgery. HRE dosing consisted of 5-10 mg/kg/day of isoniazid with no more than 300 mg/day, 5-10 mg/kg/day of rifampicin with no more than 300 mg/day, and 15 mg/kg/day of ethambutol with no more than 500 mg/day. Prudently, all patients were performed by halo traction with a weight of 0.5-1 kg preoperatively. The procedure was carried out when ESR and temperature returned to normal or significantly decreased, and when anemia and hypoproteinemia were rectified completely.

### Surgical technique

Surgery was performed under general endotracheal anesthesia. The posterior procedure was done first and then the anterior procedure done. Posterior approach: Patients were placed in the prone position with halo traction with a 1 kg/10 kg body weight during the operation. After routine exposure, posterior fixation with bilateral lateral mass plates and screws was performed in all patients. The technique for the placement of lateral mass screws was described by Magerl [13]. Instrumented fusion should reach at least the upper and lower fusion vertebrae. Fixation segments should be further extended when there is potential instability. Kyphosis correction can be attempted by posterior fixation, but cannot be done excessively; because the oppression in front of the spinal cord has not been lifted. Then, the fixation is locked. In addition to instrumented fixation, a posterolateral fusion was performed in all cases using a high-speed burr to decorticate the bilateral facet joints. Allograft particles were then packed into the decorticated facet joints. Finally, the halo traction was removed, and the wound was closed in the usual manner.

Anterior approach: If the patient’s general physical condition (cardiopulmonary function, nutritional status, etc.) can tolerate this procedure, a one-stage anterior operation should be advocated. Otherwise, a second stage surgery performed after 1–2 weeks would be implemented. In the supine position, a standard Smith-Robinson approach was used to expose the anterior cervical spine through a right-sided transverse skin incision. After routine exposure, necrotic tissue in the disc and vertebral bodies were cleaned up by curettes and pituitary forceps. Then, the paravertebral abscess was identified and drained. Limited rather than radical debridement is recommended; and during curettage, utmost care should be taken to avoid additional growth plate damage. After excision of the necrotic disc and collapsed vertebrae, a suitable flush tube was inserted into the paravertebral space or underneath the musculus longus colli to flush with a mixture of isoniazid plus saline until no pus could be retrieved. After corpectomy and decompression, cartilage endplates of the upper and lower vertebrae were excised with a cutting burr and curette; and the bony endplates were preserved. Gradual distraction was carried out using an intervertebral body spreader between the adjacent normal vertebrae to correct the prior kyphosis. The operating table was gradually extended to assist the deformity correction. Predictively, we precontoured and shaped the lordotic titanium mesh cages (TMCs) to fit the bony endplates of the upper and lower vertebrae. The TMCs were filled with allograft bone particles and placed with slight distraction to the cervical spine, followed by the release of distraction; allowing compression across the TMCs. The host bone cortical lips and preprocessed rigid posterior instrumentation act as a buttress, preventing TMCs displacement. Following TMC placement, the anterior plate was fastened by nails into the adjacent vertebrae. Patients were treated with 1.0 g of streptomycin and 0.2 g of isoniazid, which was locally administered. Then, the drainage was closed and incision sutures were performed postoperatively. The debrided material was sent for culture and histopathologic examination.

In particular, we avoided to excessively correcting the kyphosis by posteriorly instrumenting and we just carried out moderate correction. Then, during the anterior surgical procedure, we tried to distract the spinal vertebras with TMC. Just because of the moderately posterior correction, which leaves some space for anterior correcting, the anterior distraction could avoid this loosening or dislodging the posterior instrumentation.

### Postoperative managements

The drainage tube was pulled out when the volume of drainage was less than 30 ml/24 hours. Patients continued with the aforementioned three-drug HRE chemotherapy orally for two months, postoperatively. Afterwards, ethambutol was discontinued and patients received nine- to twelve-month regimens of HR chemotherapy (2HRE/9-12HR). All patients were clinically and radiologically examined at one week and three, six and twelve months after surgery, and then once a year. After two days of post-surgery, patients were allowed to ambulate with a hard cervical collar for an average of at least three months, postoperatively. Routine postoperative medical treatments were carried out including dehydration treatment, conventional nerve nutrition, and prophylactic antibiotic administration. In addition, functional rehabilitation exercise was advised to all patients during the early stage of recovery.

### Outcome evaluation and statistical analysis

Average follow-up period for all cases was 34.3 ± 8.6 months (24–48 months). The following indexes were recorded preoperatively, postoperatively and during follow-up: (1) kyphosis angle, two lines were drawn while observing the lateral X-ray (one through the superior surface of the first normal vertebra cephalic to the lesion and one through the inferior surface of the first normal vertebra caudal to the lesion), perpendiculars were drawn from these lines, and the angle was measured at their intersection; (2) visual analogue scale scores of pain; (3) ESR; and (4) body weight. Using SPSS 19.0 software, kyphosis angle, visual analogue scale scores, ESR and body weight were statistically analyzed by an independent-samples *t*-test preoperatively, postoperatively and/or during follow-up. Neurological function was statistically analyzed by Wilcoxon signed-rank test preoperatively and during follow-up. Discrepancy of the normal distribution was analyzed by rank-sum test with a significance level of 0.05. Results are reported as mean ± standard deviation (SD, Table 2).

## Results

Blood loss was 70–450 ml with a mean blood loss of 215.0 ± 102.0 ml, operation time was 85–180 min with a mean operation time of 114.6 ± 36.0 min, and hospitalization time was 5–20 days with a mean hospitalization time of 10.8 ± 5.1 days. Histopathology examination of the operative specimen revealed granulomatous infections that are consistent with tuberculosis. Average pretreatment ESR was 49.8 ± 13.2 mm/h (23 to 68 mm/h), which normalized within three months in all patients. There was a statistical difference between preoperative ESR and ESR at three months follow-up (*P* = 0.000, Table 2).

Wounds healed without chronic infection or sinus formation. Three patients revealed symptoms of mild dysphagia postoperatively, but these symptoms disappeared after patients were advised to take a liquid diet. Further, three children were complicated with dysphagia or dyspnea by retropharyngeal abscess preoperatively, but had immediate postoperative relief. Two children suffered from non-active pulmonary tuberculosis and mild tuberculous pleuritis preoperatively, which healed without any particular therapy except for anti-tuberculosis therapy of CST. Another patient was complicated with moderate tuberculosis pleural effusion preoperatively. Additional thoracocentesis plus closed drainage was performed, which harvested a satisfied outcome (Fig. 1e). Four patients had constitutional symptoms such as mild fever and night sweats, which improved after one week postoperation. Body weight of all patients significantly increased and their appetite improved during the three months of postoperative follow-up. No complications related to instrumentation and bone graft occurred.

Neurologic deficits in seven patients improved at the final follow-up examination. Results were evaluated based on the American Spinal Injury Association scoring system. At the last follow-up, among the two patients with preoperative grade B deficits, one patient recovered to grade D and the other patient returned to normal. Among the three patients with preoperative grade C deficits, two cases were classified as grade D and all patients recovered to grade E (Table 2). One child revealed incomplete neurological function (grade D) postoperatively, which attributed to delayed diagnosis. Eight patients with cervical radiculopathy had immediate postoperative remission. All patients had neck pain relief. Average preoperative visual analogue scale was 6.6 ± 1.6, which decreased to 0.3 ± 0.5 at the final follow-up (*P* = 0.000, Table 2).

**Fig. 3:**
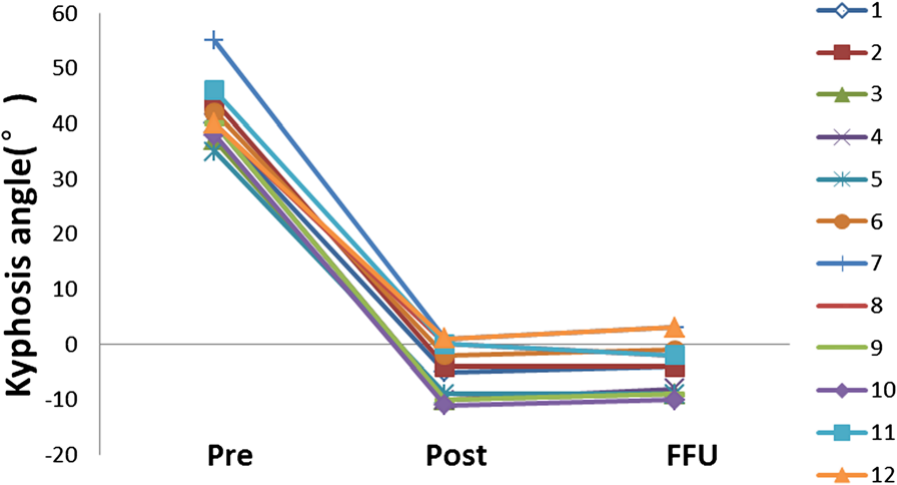
Correction of kyphosis and loss of correction in all patients. A line chart shows changes of the kyphosis angle of every patient preoperatively, postoperatively, and at final follow-up; and there was a statistiacally significant difference (*P* < 0.05). At the final follow-up, two patients (no. 8 and no. 11) improved by two degrees, rather than losing the correction. Lines with the same color represent the same patient

Kyphosis angle was 35–55° with a mean kyphosis angle of 41.4 ± 5.2°, preoperatively. This significantly decreased to -11–1° with a mean kyphosis angle of -4.9 ± 4.9°, postoperatively (*P* = 0.000). Kyphosis angle was -10-3° with a mean kyphosis angle of -4.3 ± 4.7° at the final follow-up, in which the correction of kyphosis and loss of correction was 47.1 ± 4.9° (39-55°) and 0.6 ± 1.4° (-2-2°), respectively. These measurements significantly improved as compared to preoperative measurements (*P* = 0.000). Interestingly, during the final follow-up, two patients improved by two degrees rather than losing the correction, compared with the kyphosis angle measured at one week postoperation (Table 2 and Fig. 3).

TMCs were filled with allograft bone particles, and posterolateral fusion was performed in all patients. Lateral and flexion-extension X-ray and CT-scans did not reveal any settling, expulsion or migration of TMCs, or loosening of lateral mass screws. The presence of continuous bridging and bone trabeculae at the graft-host vertebral endplate junction was identified by CT-scans (Fig. 2). Pseudoarthrosis was determined by the absence of osseous trabecular bridging between the graft and host vertebra endplates and the presence of a lucent line at the graft-vertebral junctions in the sagittal reconstruction CT scans [14]. In our study, bone healing was achieved in all patients after a mean period of 5.4 months; which were confirmed by two different surgeons based on the above criteria of radiological fusion.

## Discussion

CST is rarely seen in children, compared with thoracic and lumbar spinal tuberculosis; which mostly occurs in 2–5 year old children [15]. CST is prone to shifts in the cervical spine sagittal alignment and necrotic tissue compression in the cervical spinal cord. If not treated, this can be complicated by paraplegia or even panplegia. Small lesions and unobvious systemic and local symptoms make early diagnosis difficult during the initial stage of pediatric CST. Some scholars have discovered vessels existing in endplate cartilage specimens of children; suggesting that children, regardless of whether spinal tuberculosis occurs first in the vertebral body or intervertebral space, are more likely to spread among different segments, compared with adults. Moreover, the connection between the vertebral periosteum and body is relatively loose. Therefore, pus between the gaps may easily spread in the surroundings. Due to wedge and collapse changes in the vertebrae following invasion, combined with relatively fast bone growth in children, growth imbalance between the anterior and posterior elements gradually produce kyphosis [16, 17]. In addition, spinal tuberculosis involves the anterior column more frequently; and spinal tuberculosis combined with damaged adjacent vertebral discs lead to anterior instability, while the posterior structure relatively remains intact. As the disease progresses, along with the forward load of the cervical spine, cervical kyphosis further intensifies. This increases tension within the spinal cord, causing vasoconstriction followed by intramedullary ischemia; resulting in spinal cord dysfunction. Unfortunately, effective anti-tuberculosis therapy and immobilization turns this uncomplicated pediatric CST into a medical disease due to the strong regeneration capacity and low drug resistance in children compared with adults [3]. Cervical kyphotic deformity and restricted head and neck activity upon admission have been evident in most children. This condition cannot be solely treated by head-neck tractions and drugs, and requires surgical treatment.

According to the above characteristics, tuberculosis elimination, removal of nerve compression, correction of kyphosis, reconstruction of cervical stability, and the restoration and maintenance of cervical lordosis have become the main purpose of the surgical treatment of PCSTK [13]. Stand-alone anterior surgery advantages for pediatric CST include improved visualization and radical debridement, which directly addresses clinically relevant pathologies and provides more extensive decompression and reconstruction of the anterior column. These advantages theoretically lead to improved rates of arthrodesis and perfect clinical outcome. Especially in recent years, the prevalent application of the cervical anterior plate can provide immediate stability and maintain intervertebral height and cervical lordosis reconstruction; which could prevent graft or interbody support sliding, increase fusion rate, and reduce the relapse of tuberculosis. However, anterior radical curettage and excision of the ring apophysis and end-plate in pediatric CST destroys anterior growth and restricts the capacity for spinal remolding; contributing to the progressive development of kyphosis during growth [10, 11, 18]. Therefore, PCSTK treatment should not only aim to cure the affection, but also aim to correct and maintain cervical stability and sagittal alignment by preventing the additional progression of bony destruction and equilibrating growth potential during the treatment period. Furthermore, graft or TMC and plate displacement has been reported in 5–50 % of patients following a stand-alone anterior stabilization procedure, especially in treating multiple-level corpectomy [19–22].

Thus, the addition of adjunctive posterior instrumentation is recommended by some surgeons [9, 12, 19, 23–26]. Richman et al. used a porcine model to evaluate cervical stability after a single-level corpectomy, and found that anterior grafting followed by posterior lateral mass plating was superior to anterior plating alone [27]. Nottmeier et al. [23] describes 41 patients with cervical kyphotic deformity, who underwent 360-degree reconstruction. They suggested that unilateral surgical approaches for treating cervical kyphotic deformity have theoretically decreased perioperative risk, but may sacrifice both the degree and maintenance of deformity correction; emphasizing the degree of deformity correction and maintenance of deformity correction using 360-degree reconstruction. The study of Jain et al. [28] revealed that progressive kyphotic deformity should be considered for kyphosis correction in an active disease or early post-treatment stage, and that posterior fusion was advocated as a method to halt posterior column growth and arrest the progression of kyphosis in PCSTK. Combined anterior and posterior approaches would significantly reduce stress on the plate and graft, increase cervical spine stability, and reduce graft and/or fixation-related complications [29]. McAfee et al. [30] reported 12 cases that obtained stable bone fusion and satisfactory kyphosis correction without dislodgement and loss of correction after undergoing titanium mesh cage fusion and circumferential reconstruction.

In the present study, three cases underwent two-stage surgery due to poor constitution and intolerance to continuous trauma. All patients achieved satisfied clinical and radiographic results, which were superior to reports by other surgeons [2, 4, 7]; in which a stand-alone anterior approach was used. We may attribute its mild superiority to the effectiveness of the auxiliary posterior rigid fixation. Interestingly, during the final follow-up, kyphosis of two patients increased by two degrees, rather than losing the correction. This was consistent with the study of Govender et al. [3], which indicated the spontaneous correction of kyphosis due to the anterior reserved remodeling potential.

Controversy exists regarding the preferred treatment of PCSTK. Thus, indications for surgery could be summarized as follows: (a) a severe kyphosis angle of more than 30° or mild kyphosis deformity with progressive instability; (b) multilevel (≥2 levels) continuous segments and anterior column severely damaged or complicated by long-segment cervical spinal stenosis; (c) significantly deformed dura mater, as well as severe or progressive neurological dysfunction and persistent neck pain or restricted neck activity unresponsive to conventional therapy (anti-tuberculosis therapy, immobilization and hyper-alimentation); (d) spinal cord compression by paravertebral/epidural abscess or retropharyngeal abscess resulting in dyspnea and/or dysphagia; and (e) the age of the child is more than six years old (Table 3). In consideration of children with CST that presented with poorer constitution, the following exclusion criteria of the present study should be stressed: (a) mono-segmental CST with mild kyphosis, which could be resolved by stand-alone anterior reconstruction and stabilization; (b) upper CST; (c) the age of the child is less than six years old.

**Table 3:** Proposed treatment algorithm in the treatment of PCSTK

Surgical approaches	Indications of surgery
Anterior	1,mild kyphosis, less than 30° and after preoperative halo traction, the kyphosis changed to less than 5-10°;
	2,single segment lesion and confined to the anterior and central column of spine;
	3,spinal cord compression by paravertebral/epidural abscess or retropharyngeal abscess resulting in dyspnea and/or dysphagia;
	4, the age less than 6 years old, and cannot suffer too much trauma.
Posterior	1,the lesion limited to the posterior column of spine and the compression to spinal cord from the posterior column ;
	2, the history of anterior surgery.
Combined Anterior and Posterior	1, severe kyphosis angle, more than 30° or mild kyphosis deformity but progressive instability;
	2, multilevel (≥2 levels) continuous segments and anterior column damaged severely or complicated by long-segment cervical spinal stenosis;
	3, the age of children more than 6 years old.

From our experience, the treatment of PCSTK should focus on the following points. (a) Avoid expanding the scope of debridement and resection of the entire vertebral body blindly, which is not conducive to the clinical repair and healing of tuberculosis. In this study, we tried to keep the remaining vertebra surrounding the focus, facilitating the TMC to fuse with circumambient bone tissues. (b) Pay attention to the abscess buried in the musculus longus colli. We repeatedly washed the pus by a mixture of isoniazid plus saline using a thin catheter, which not only thoroughly eliminated the abscess, but also markedly increased the local concentration of anti-TB drugs. (c) A negative tuberculin test, PPD test, or polymerase chain reaction should not exclude the consideration of *M. tuberculosis*. Plain radiographic changes may lag for many months behind pathological alterations, and constitutional symptoms do not contribute to the diagnosis of PCSTK in most patients in our series. As a result, early diagnosis may depend on MRI or CT scans, and microscopy and culture of the infected material are recommended for the final diagnosis of CST. (d) Standard chemotherapy at the early stage of the disease is beneficial in minimizing complications and promoting early recovery. Most importantly, directly observed therapy is a vital strategy for interrupting the recurrence of tuberculosis disease; which should be implemented for all patients. (e) Pediatric CST is often merged with pulmonary tuberculosis and/or tubercular pleural effusion. If not combined with open tuberculosis, surgery could be generally applied for patients; and conventional anti-TB treatment of PCSTK could cure pulmonary tuberculosis or mild pleural effusion simultaneously. However, severe pleural effusion requires thoracocentesis and closed chest drainage afterwards. (f) Supportive care should be emphasized. Young patients are vulnerable to malnutrition, anemia and hypoalbuminemia due to long-term anorexia and insufficient sleep caused by persistent neck pain. Preoperative immobilization, nutrition support, sedative analgesia, multiple low doses of albumin, and blood transfusion during the perioperative period are helpful [31]. (g) 360-degree instrumented and TMC bone fusion can rigidly provide temporary and long-term stability. Therefore, the patient does not need to suffer immobilization in bed after the operation, and rehabilitation exercises should be encouraged after two days of postoperation [32, 33]. (h) With regard to the optimum time for removing implants, we maintained the implants in patients in this study. Patients would suffer further kyphosis if fixations are moved too early due to the remolding ability of the pediatric spine during growth. In practice, we choose to remove the implants when growth was terminated; and perform frequent follow-ups. However, all patients in our study continued to have significant growth, and the most significant limitations of this study were the study’s retrospective nature, small sample size, and the relatively short follow-up. Therefore, prospective and larger studies with longer follow-up periods are needed.

## Conclusions

This present study revealed that 360-degree fusion combined with anterior debridement and decompression should have a beneficial influence on pediatric cervical spinal tuberculosis and segment, as well as global spinal reconstruction, correction and maintenance of kyphosis and bone fusion. Anterior debridement and bone fusion is a prerequisite in the surgical treatment of PCSTK. However, it should always be accompanied by posterior fixation and fusion to shorten the immobilization period, obtain a good and long lasting correction of kyphosis, and prevent further collapse and graft failure, especially for children with kyphosis.

## Ethics approval and consent to participate

Written informed consent was obtained from all guardians of patients. This study protocol was approved by the Ethics Committee of Xiangya Hospital.

## Availability of data and materials

All datasets on which the conclusions of the manuscript rely were presented in the main paper.

## Abbreviations

CST: cervical spinal tuberculosis
CT: computed tomography
ESR: erythrocyte sedimentation rate
HRE: Isoniazid (H), Rifampicin (R), Ethamutol (E)
MRI: magnetic resonance imaging
PCSTK: pediatric cervical spinal tuberculosis with kyphosis
TMC: titanium mesh cages
